# Antibody-Mediated Protection against *Plasmodium* Sporozoites Begins at the Dermal Inoculation Site

**DOI:** 10.1128/mBio.02194-18

**Published:** 2018-11-20

**Authors:** Yevel Flores-Garcia, Gibran Nasir, Christine S. Hopp, Christian Munoz, Amanda E. Balaban, Fidel Zavala, Photini Sinnis

**Affiliations:** a Department of Molecular Microbiology & Immunology, Johns Hopkins Bloomberg School of Public Health, Baltimore, Maryland, USA; University of California Los Angeles

**Keywords:** antibodies, malaria, preerythrocytic, skin, sporozoites, vaccine

## Abstract

Plasmodium sporozoites are injected into the skin as mosquitoes probe for blood. From here, they migrate through the dermis to find blood vessels which they enter in order to be rapidly carried to the liver, where they invade hepatocytes and develop into the next life cycle stage, the exoerythrocytic stage. Once sporozoites enter the blood circulation, they are found in hepatocytes within minutes. In contrast, sporozoite exit from the inoculation site resembles a slow trickle and occurs over several hours. Thus, sporozoites spend the majority of their extracellular time at the inoculation site, raising the hypothesis that this is when the malarial parasite is most vulnerable to antibody-mediated destruction. Here, we investigate this hypothesis and demonstrate that the neutralizing capacity of circulating antibodies is greater at the inoculation site than in the blood circulation. Furthermore, these antibodies are working, at least in part, by impacting sporozoite motility at the inoculation site. Using actively and passively immunized mice, we found that most parasites are either immobilized at the site of injection or display reduced motility, particularly in their net displacement. We also found that antibodies severely impair the entry of sporozoites into the bloodstream. Overall, our data suggest that antibodies targeting the migratory sporozoite exert a large proportion of their protective effect at the inoculation site.

## Abstract (precis)

Studies in experimental animal models and humans have shown that antibodies against Plasmodium sporozoites abolish parasite infectivity and provide sterile immunity. While it is well documented that these antibodies can be induced after immunization with attenuated parasites or subunit vaccines, the mechanisms by and location in which they neutralize parasites have not been fully elucidated. Here, we report studies indicating that these antibodies display a significant portion of their protective effect in the skin after injection of sporozoites and that one mechanism by which they work is by impairing sporozoite motility, thus diminishing their ability to reach blood vessels. These results suggest that immune protection against malaria begins at the earliest stages of parasite infection and emphasize the need of performing parasite challenge in the skin for the evaluation of protective immunity.

## INTRODUCTION

Malaria remains one of the most important infectious diseases in the world, causing significant morbidity and mortality, particularly in resource-poor settings. Plasmodium parasites, the causative agents of malaria, cycle between mosquito and mammalian hosts. In the mammalian host, infection has two distinct phases, an asymptomatic preerythrocytic stage when parasite numbers are low, and a symptomatic erythrocytic stage responsible for all clinical symptoms of the disease. Efforts to generate a malaria vaccine have focused on both of these stages, with vaccine candidates targeting sporozoites demonstrating some promise (1). Though short-lived in the mammalian host, their low numbers and extracellular residence time likely make them more susceptible than other life cycle stages to the effect of antibodies. Indeed, the protection observed in human vaccine recipients closely correlates with antibody titers against sporozoites (2–4).

Sporozoites are the infective stage of the malarial parasite and must make a remarkable journey from the site at which they are deposited by infected mosquitoes to the liver, where they invade hepatocytes and transform into the next life cycle stage. This is a bottleneck for the parasite, with 10 to 100 sporozoites being inoculated (5) and only a fraction ultimately making it to the liver and developing to mature liver-stage parasites (6, 7). The barriers faced by sporozoites are only beginning to be appreciated, with the first hurdle being exit from the inoculation site. Several lines of evidence demonstrate that sporozoites are deposited into the skin and not directly into the blood circulation, including direct visualization of the process by intravital imaging (5–9). After their inoculation, sporozoites actively move in the skin to find and penetrate blood vessels to enter the blood circulation and be transported to the liver (6, 7). Like all apicomplexan parasites, sporozoites move by a substrate-based motility called gliding motility, powered by an actin-myosin motor beneath the plasma membrane (10). Plasmodium sporozoites are faster and move for longer periods of time than other Plasmodium life cycle stages, suggesting that their fast robust motility may have evolved for exit from the inoculation site. Indeed, this notion is supported by the phenotype of two motility mutants, a thrombospondin-related anonymous protein (TRAP) mutant that moves more slowly (11) and a deletion mutant of TRAP-like protein (TLP [12]). Both mutants are significantly more attenuated in their ability to cause infection, after inoculation into the skin, thus highlighting the role of sporozoite motility in exit from the inoculation site.

Investigation into the kinetics with which sporozoites exit the inoculation site revealed that although some sporozoites leave within minutes, many take 30 to 120 min to exit (13). These data from experiments with rodent malaria parasites are supported by studies in humans and monkeys. In monkeys, transplantation of the dermal bite site 2 h after the bites of Plasmodium cynomolgi-infected mosquitoes resulted in infection in naive recipients (14). Additional experiments in humans that were fed upon by Plasmodium vivax- and Plasmodium falciparum-infected mosquitoes showed that blood removed from these subjects 1 h post-mosquito bite could initiate malaria infection in naive recipients (15). In contrast to the time it takes for sporozoites to transit from the dermis to the bloodstream, once in the blood circulation, sporozoites are arrested in the liver and enter hepatocytes within minutes (13, 16, 17). Thus, for sporozoites, the inoculation site is where the parasite is extracellular for the longest period of time and therefore likely to be most vulnerable to antibody-mediated neutralization.

An efficacious malaria vaccine would significantly contribute to the control and possibly elimination of malaria. Early studies showed that immunization of birds and mice with radiation-attenuated sporozoites conferred protection, and this model has served as the gold standard for preerythrocytic-stage vaccine candidates (18, 19). Follow-up studies demonstrated that antibodies targeting the major surface protein of sporozoites, the circumsporozoite protein (CSP), and T cells specific for infected hepatocytes were the basis of this protection (20, 21). These studies led to the development of a CSP-based subunit vaccine candidate called RTS,S. Phase III clinical trials of RTS,S demonstrated 40% to 50% efficacy in preventing clinical disease for 1 year, with protection waning significantly at later time points (2). Though there is need for improvement, this is a milestone for the malaria vaccine field and validates the sporozoite and its major surface proteins as targets. Follow-up studies of protected and unprotected children participating in this trial demonstrated that antibody titers correlate with protection (3, 4). We believe that the low sporozoite inoculum together with the length of time the parasite is extracellular at the inoculation site make the migratory sporozoite the most vulnerable Plasmodium life cycle stage in the mammalian host. In this study, we use the rodent malaria model to investigate the effect of circulating anti-sporozoite antibodies on sporozoite motility and infectivity at the dermal inoculation site.

## RESULTS

### Sporozoites have impaired movement in the skin of immunized mice.

**FIG 1:**
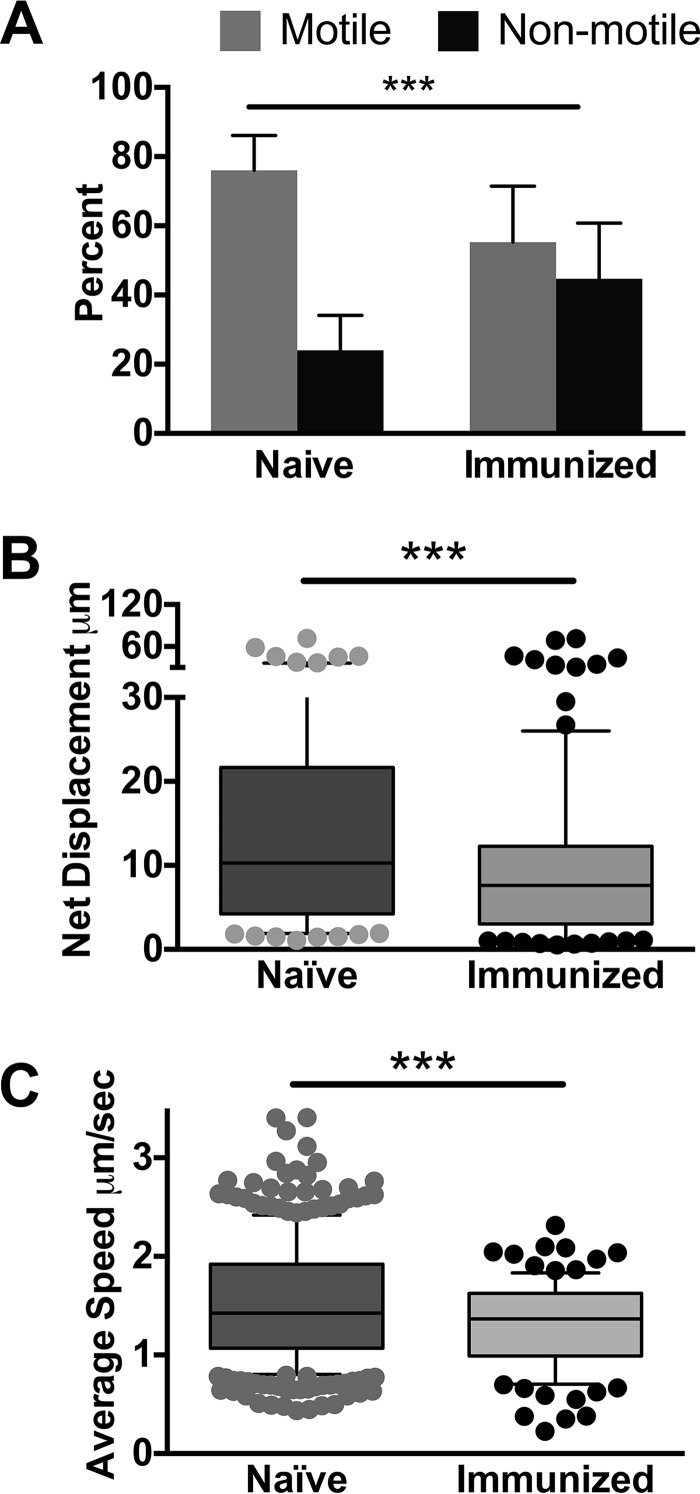
Sporozoites inoculated into the skin of irradiated-sporozoite immunized wild-type mice exhibit impaired motility. Wild-type mice were immunized with irradiated *P. berghei* sporozoites. Three weeks after the last boost, *P. berghei* mCherry sporozoites were inoculated into immunized or naive mice and imaged 10 min after inoculation by confocal microscopy. (A) Motile and nonmotile sporozoites were manually counted, and shown is the percent motile and nonmotile sporozoites in immunized and naive control mice. Data from 3 independent experiments were pooled and are shown are means ± standard deviations. The proportions of motile to nonmotile sporozoites in naive and immunized mice are significantly different by Fisher’s exact test (*P* < 0.001). (B and C) Sporozoite movement in the skin of immunized and naive mice was recorded at 10 min after sporozoite inoculation, and trajectories were analyzed. Shown are net displacement (B) and average speed (C) of motile sporozoites over the entire length of the movie. Movies from 3 independent experiments were analyzed, and shown is a representative experiment. Statistical comparisons of net displacement and speed in naive and immunized mice were performed using a linear mixed-effects model on the pooled data. Data from all 3 experiments are shown in Fig. S2. ***, *P* < 0.001.

With the knowledge that sporozoites spend some time at the inoculation site and must be motile to find and enter blood vessels, we used the rodent model to investigate the impact of immunization on sporozoite motility at the inoculation site. We immunized mice with radiation-attenuated Plasmodium berghei sporozoites, and 3 weeks after the last immunization, when mice are protected from challenge with live sporozoites (see Fig. S1 in the supplemental material), we performed intravital imaging of P. berghei sporozoites expressing mCherry in immunized and age-matched naive mice. Sporozoites were inoculated into the ear pinna of mice, and their motility was visualized in 5-min movies, beginning 10 min post-inoculation. We found that sporozoites inoculated into the skin of immunized mice display significantly altered motility compared to sporozoites inoculated into the skin of naive mice (Fig. 1A to C). Significantly higher numbers of sporozoites were not moving in immunized mice at 10 min after inoculation (Fig. 1A). With those sporozoites that were motile, we quantified their displacement and speed in immunized and naive mice. Net displacement, defined as the distance along a straight line between the initial and final positions of motile sporozoites over the duration of a 5-min movie, was significantly reduced in sporozoites in immunized mice (Fig. 1B). Quantification of the average speed of each sporozoite over the course of 5-min movies showed that motile sporozoites in immunized mice also had a reduced average speed (Fig. 1C). Interestingly, the reductions in sporozoite displacement and speed in immunized mice were largely in the upper quartiles; the distances and speeds reached by the upper quartiles of moving sporozoites were significantly lower in immunized mice. (A representative experiment is shown in Fig. 1B and C, and results from all 3 biological replicates are shown in Fig. S2). Overall, these data suggested that immune responses generated by immunization with radiation-attenuated sporozoites impact parasite motility at the inoculation site.

10.1128/mBio.02194-18.1 FIG S1 Liver parasite burden of sporozoite-immunized and naive control mice. Mice were immunized with irradiated *P. berghei* sporozoites, and 3 weeks after the last boost, immunized and control mice were challenged with 2,000 *P. berghei* sporozoites inoculated intravenously. Forty hours post-sporozoite inoculation, livers were harvested, and parasite burden was quantified by RT-qPCR, using a standard curve of *P. berghei* 18S rRNA plasmid. There are 4 to 5 mice per group, and shown is the mean ± standard deviation. The difference in liver parasite burden is significantly different between naive and immunized mice (*P* < 0.05, *P* = 0.0159), Mann-Whitney test. Download FIG S1, DOCX file, 0.03 MB. Copyright © 2018 Flores-Garcia et al. 2018 Flores-Garcia et al. This content is distributed under the terms of the Creative Commons Attribution 4.0 International license.

10.1128/mBio.02194-18.2 FIG S2 Net displacement and speed of sporozoites inoculated into the skin of sporozoite-immunized mice. Mice were immunized with irradiated *P. berghei* sporozoites, and 3 weeks after the last boost, *P. berghei* mCherry sporozoites were inoculated into the ear pinna and imaged 10 min after inoculation. Shown are net displacement (top) and average speed (bottom) of motile sporozoites over the length of 5-min movies from 3 independent experiments. Statistical comparisons were performed using a linear mixed-effects model on the pooled data and for both net displacement and speed; the differences between naive and immunized mice are statistically significant (*P* < 0.001). Download FIG S2, DOCX file, 0.1 MB. Copyright © 2018 Flores-Garcia et al. 2018 Flores-Garcia et al. This content is distributed under the terms of the Creative Commons Attribution 4.0 International license.

**FIG 2:**
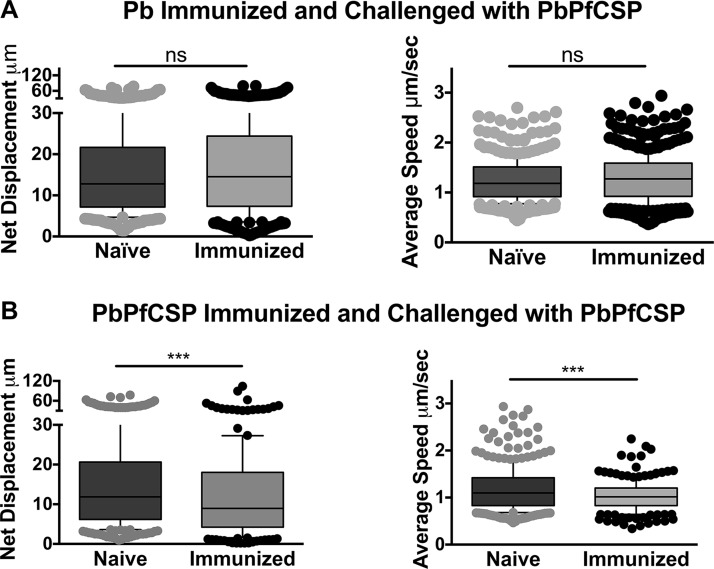
Mice were immunized with irradiated *P. berghei* (Pb) or *P. berghei* expressing *P. falciparum* CSP (PbPfCSP) sporozoites, and 3 weeks after the last boost, PbPfCSP sporozoites were inoculated into naive and immunized mice and imaged at 10 min post-inoculation. Videos were recorded and sporozoite motility was analyzed. Shown are net displacement and average speed of motile sporozoites over the entire length of the movie. Movies from 2 independent experiments were pooled. Statistical comparisons were performed using a linear mixed-effects model. ***, *P* < 0.001; ns, not significant.

We hypothesized that the inhibitory effect on sporozoite motility in immunized mice was due to antibodies targeting the major surface protein of sporozoites, the circumsporozoite protein (CSP). Indeed, previous studies have shown that antibodies to CSP are a prominent component of the immune response observed after immunization with irradiated sporozoites (22). Furthermore, antibodies specific for CSP can confer protection and have been shown to immobilize sporozoites *in vitro* (20, 23). To determine if the antibody response that impacted sporozoite motility after immunization with irradiated sporozoites was specific to CSP, we generated a fluorescent P. berghei parasite in which the endogenous *csp* gene was replaced by the *csp* coding sequence from the human malaria parasite Plasmodium falciparum (PbPfCSP; Fig. S3). Importantly, antibodies to P. falciparum CSP do not cross-react with P. berghei CSP, and vice versa (Fig. S3). Transfections were performed in the P. berghei mCherry line used for the intravital imaging experiments, as this line does not contain a selection cassette and can therefore be used to generate fluorescent mutant or transgenic lines (7). Transgenic PbPfCSP parasites develop normally in the mosquito and display normal infectivity in mice (Fig. S3). To look at the role of CSP-specific antibodies on sporozoite motility in the skin, mice were immunized with P. berghei sporozoites, and following immunization, the motility of PbPfCSP sporozoites in immunized and naive mice was assessed by confocal microscopy. In contrast to the significant differences in net displacement and average speed of wild-type P. berghei sporozoites (Fig. 1B and C), we found no significant difference in either parameter when PbPfCSP mCherry parasites were inoculated into P. berghei-immunized mice (Fig. 2, top). In contrast, when mice were immunized with PbPfCSP parasites, net displacement and speed of PbPfCSP sporozoites were significantly decreased (Fig. 2, bottom). Taken together, sporozoite immunization impacts the motility of sporozoites at the inoculation site and appears to be mostly directed against the CS protein of sporozoites. This is not unexpected, considering the well-documented strong immunodominance of CSP (24).

10.1128/mBio.02194-18.3 FIG S3 Generation and verification of a *P. berghei* parasite expressing *P. falciparum* CSP. (A) Plasmid pR-CSPfFL (35) containing the *P. falciparum csp* coding region with a *P. berghei* signal sequence, the hDHFR selection cassette, and *csp* flanking regions to guide insertion into the *csp* locus was transfected into mCherry expressing *P. berghei* parasites (507cl1 [34]) to yield *P. berghei* mCherry parasites in which the endogenous *csp* gene was replaced by the full-length *P. falciparum* CSP. Xs mark homology areas for recombination; restriction sites are abbreviated as follows; X, XhoI; K, KpnI; Ka, KasI; P, PmlI; B, BsmFI. (B) PCR verification of the *csp* gene in the transfected *P. berghei* mCherry expressing Pf CSP (Pb/Pf) and the parental *P. berghei* mCherry line (WT). PCR was performed with genomic DNA of each parasite line with primers specific for *P. berghei csp* and *P. falciparum csp,* as indicated. (C) Salivary gland sporozoite numbers in the parental *P. berghei* mCherry line (Pb) and the transfected *P. berghei* mCherry expressing Pf CSP (Pb/PfCSP). Salivary glands from 20 infected mosquitoes were harvested and homogenized, and sporozoites were counted. Shown is the mean number of salivary gland sporozoites for each parasite line. (D) Sporozoite infectivity in C57Bl/6 mice. Mice were inoculated intravenously with 2,000 *P. berghei* mCherry (Pb) or *P. berghei* mCherry expressing Pf CSP (PbPfCSP) sporozoites, and 40 h later, parasite liver load was quantified by RT-qPCR. There were 5 mice per group, and shown is the mean ± standard deviation for each group. (E) Immunofluorescence analysis of CSP expression. *P. berghei* mCherry and *P. berghei* mCherry expressing PfCSP sporozoites were harvested from mosquito salivary glands, fixed, and stained with monoclonal antibodies specific for *P. berghei* CSP (MAb 3D11) or *P. falciparum* CSP (MAb 2A10). Shown are representative images. Download FIG S3, DOCX file, 7.2 MB. Copyright © 2018 Flores-Garcia et al. 2018 Flores-Garcia et al. This content is distributed under the terms of the Creative Commons Attribution 4.0 International license.

### Sporozoites have impaired motility in the skin of passively immunized mice.

**FIG 3:**
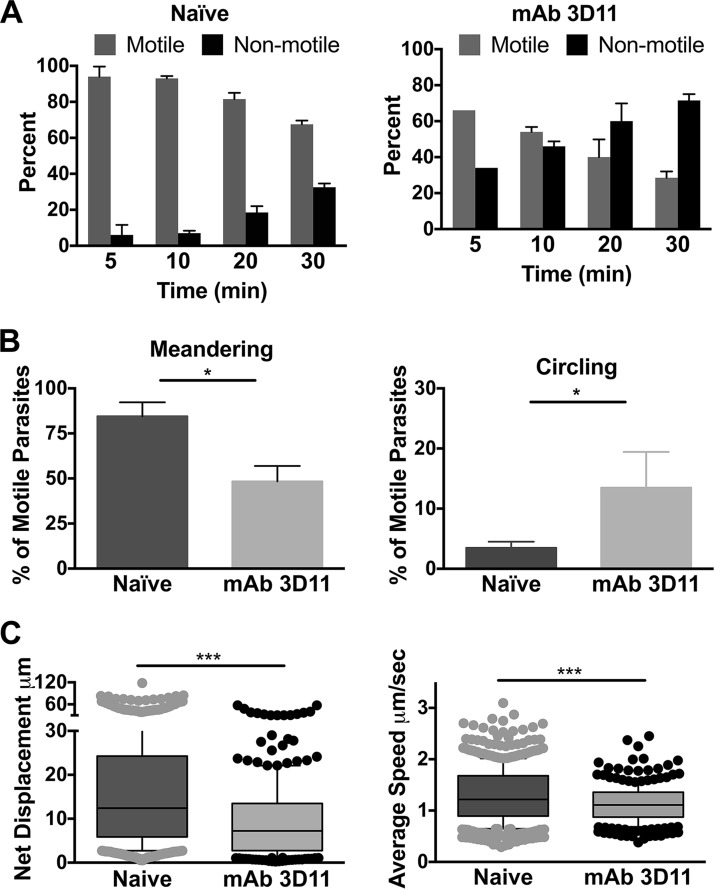
Sporozoites inoculated into the skin of mice passively immunized with MAb 3D11 exhibit impaired motility. Mice were passively immunized by i.v. inoculation of 150 µg of MAb 3D11. Sixteen hours later, *P. berghei* mCherry sporozoites were injected intradermally, and their movement in the skin was recorded in 5-min videos and analyzed. (A) Motile and nonmotile sporozoites were manually counted at the indicated time points after sporozoite inoculation, and shown are the percentages of motile and nonmotile sporozoites in MAb 3D11-immunized and naive control mice at each time point. At least 100 sporozoites were imaged per movie per time point. Results from 2 time courses were pooled, and shown is the mean ± standard deviation. The proportions of motile to nonmotile sporozoites between naive and MAb 3D11 inoculated mice were statistically significantly different at all time points (Fisher’s exact test, *P* < 0.001). (B) The nature of sporozoite trajectories at 10 min post-sporozoite inoculation into control and passively immunized mice was determined. Shown is the percentage of motile sporozoites that were exhibiting more linear meandering trajectories (left) or circular trajectories (right). Shown is the mean ± standard deviation of 3 biological replicates. *, *P* < 0.05 by the Mann-Whitney test. (C) Net displacement and speed of motile sporozoites in passively immunized and naive control mice at 10 min post-sporozoite inoculation measured over the entire length of the 5-min movie. Three independent experiments were performed, and shown is a representative experiment. Statistical comparisons of net displacement and speed in naive and immunized mice were performed using a linear mixed-effects model on the pooled data. Data from all 3 experiments are shown in Fig. S4. ***, *P* < 0.001.

CS proteins of all Plasmodium species have a central repeat region the sequence of which varies among species. This region is immunodominant and is recognized as the target of the majority of antibodies generated by immunization with irradiated sporozoites (22). Importantly, monoclonal antibodies generated from immunized mice and targeting the CSP repeats phenocopy the effect of sporozoite immunization, inhibiting sporozoite infectivity *in vivo* (22) and impacting motility *in vitro* (23). To better characterize the effect of CSP-specific antibodies on sporozoite motility at the inoculation site, we passively immunized mice with 150 µg of monoclonal antibody (MAb) 3D11, specific for the repeat region of P. berghei CSP (20). This antibody concentration is considered to be partially protective in humans vaccinated with the P. falciparum CSP vaccine RTS,S (2). Sixteen hours after intravenous inoculation of MAb 3D11, P. berghei mCherry sporozoites were inoculated intradermally into naive and passively immunized mice and visualized by confocal microscopy. We first quantified the number of motile and nonmotile sporozoites over time in passively immunized and control mice. In both groups, the proportion of nonmotile sporozoites increased over time. However, while in naive mice the nonmotile population went from 5.6% to 30% over a 30-min time period, in passively immunized mice, 28% of sporozoites were not motile at 5 min, and this increased to 70% by 30 min (Fig. 3A). At each time point, there were significantly more nonmotile sporozoites in mice passively immunized with MAb 3D11 than in the controls.

10.1128/mBio.02194-18.4 FIG S4 Net displacement and speed of sporozoites inoculated into the skin of naive and passively immunized mice. Mice were passively immunized by i.v. inoculation of 150 μg of MAb 3D11, and 16 h later, *P. berghei* mCherry sporozoites were inoculated into the ear pinna of naive and immunized mice and imaged 10 min after inoculation. Shown are net displacement (top) and average speed (bottom) of motile sporozoites over the length of 5-min movies from 3 independent experiments. Statistical comparisons were performed using a linear mixed-effects model on the pooled data, and for both net displacement and speed, the differences between naive and immunized mice are statistically significant (*P* < 0.001). Download FIG S4, DOCX file, 0.2 MB. Copyright © 2018 Flores-Garcia et al. 2018 Flores-Garcia et al. This content is distributed under the terms of the Creative Commons Attribution 4.0 International license.

Following this, we evaluated the trajectories of motile sporozoites in passively immunized and naive control mice. A previous study found that sporozoites initially move in more linear paths, maximizing the tissue volume they explore, and by 30 min post-inoculation, their trajectories become more constrained, a pattern that may optimize interactions with blood vessels (7). In passively immunized mice, we observed lower numbers of sporozoites moving linearly with an enhanced proportion of circling sporozoites than sporozoites inoculated into naive mice (Fig. 3B). Last, similar to what we observed in mice immunized with radiation-attenuated sporozoites, both net displacement and speed of motile sporozoites were significantly reduced in passively immunized mice compared to those in naive mice (Fig. 3C and S4). Interestingly, active and passive immunization impacted sporozoites similarly, with the reductions in displacement and speed being most clearly observed in the upper quartile values.

### Passive immunization with lower doses of MAb 3D11 impacts sporozoite motility.

**FIG 4:**
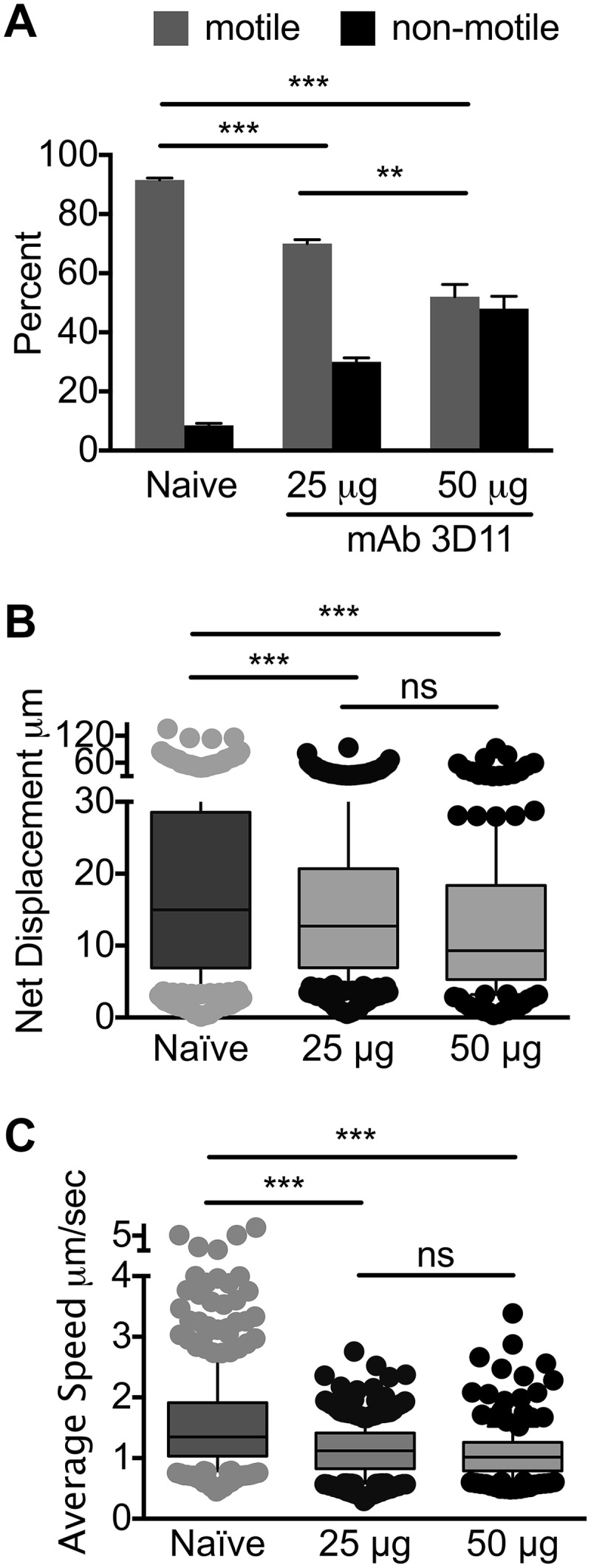
Lower doses of mAb 3D11 impact sporozoite motility at the inoculation site. Mice were passively immunized by i.v. inoculation of either 25 µg or 50 µg of MAb 3D11, and 16 h later, *P. berghei* mCherry sporozoites were injected intradermally and imaged by confocal microscopy at 10 min post-sporozoite inoculation. (A) Motile and nonmotile sporozoites were manually counted, and shown are the percentages of motile and nonmotile sporozoites in immunized and naive control mice. Data from 2 independent experiments were pooled, and shown is the mean ± standard deviation. The proportion of motile to nonmotile sporozoites between naive and 25 µg or 50 µg MAb 3D11 was statistically significant, as was the difference between 25 µg and 50 µg MAb 3D11 (Fisher’s exact test, ***, *P* < 0.01). (B and C) Sporozoite trajectories were analyzed; shown is the net displacement (B) and speed (C) of motile sporozoites over the entire length of the 5-min movies. Data from two biological replicates were pooled, and statistical analysis was performed using a linear mixed-effects model. ***, *P* < 0.001; ns, not significant.

Though the dose of MAb 3D11 we used is in keeping with anti-CSP titers observed in RTS,S and irradiated sporozoite-vaccinated individuals (25–27), this is a monoclonal antibody and not polyclonal serum and perhaps more potent than the antibodies generated by RTS,S vaccination. We therefore tested lower doses, 50 and 25 µg, of MAb 3D11 for their impact on sporozoite motility at the inoculation site, as outlined above. As shown in Fig. 4A, passive immunization with 25 and 50 µg of MAb 3D11 significantly reduced the number of motile sporozoites in a dose-dependent manner. Furthermore, analysis of the motile sporozoites showed significantly reduced net displacement and speed in passively immunized mice for both concentrations of 3D11 compared to naive mice; however, in this case, there was no difference in the inhibition observed between 25 and 50 µg of MAb 3D11 (Fig. 4B and C).

### Sporozoites inoculated into passively immunized mice exhibit significantly reduced blood vessel invasion.

**FIG 5:**
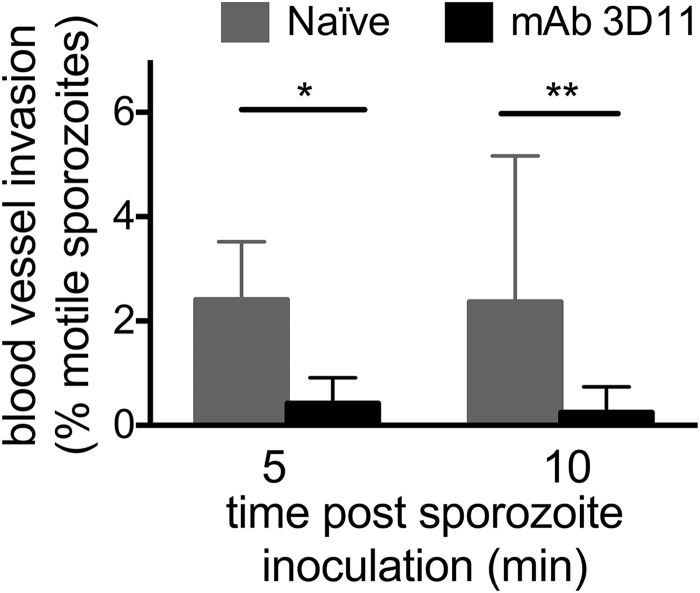
Sporozoites inoculated into passively immunized mice exhibit decreased blood vessel invasion. Mice were passively immunized by i.v. inoculation of 150 µg of MAb 3D11, and 16 h later, *P. berghei* sporozoites expressing mCherry were injected intradermally and imaged by confocal microscopy. Five-minute movies were recorded at the indicated time points, and the percentage of motile sporozoites entering into the blood circulation was scored. Data were pooled from 4 videos per condition per time point, and shown is the mean ± standard deviation of the percentage of motile sporozoites that entered blood vessels. Statistical analysis was performed using Fisher’s exact test on the pooled data. *, *P* < 0.05; **, *P* < 0.01.

We next evaluated the effect of passive immunization with MAb 3D11 on the ability of sporozoites to invade blood vessels, a critical step for the parasite to reach the liver and establish infection. Our previous study suggested that approximately 20% of the inoculum succeeds in finding and entering blood vessels, with approximately 2 to 4% of motile sporozoites entering blood vessels in any single 4-min movie recorded between 5 and 30 min post-inoculation (7). In this study, blood vessel invasion events were scored at 5 and 10 min post-sporozoite inoculation in mice passively immunized with 150 μg of MAb 3D11 and in naive control mice. In naive mice, the frequency of blood vessel invasion is concordant with those in previously published studies (6, 7). In contrast, the rate of blood vessel invasion is significantly reduced at both time points in passively immunized mice (Fig. 5). This reduction is likely due to the lower number of motile sporozoites, combined with the decrease in displacement and speed of those that are motile.

### Passively transferred antibody is more protective against mosquito bite-inoculated sporozoites.

Thus far, our data suggest that antibody targeting sporozoites, either passively administered or generated by active immunization, has a significant impact on sporozoite motility in the skin. Given that motility is critical for sporozoite exit from the inoculation site and entry into the bloodstream, we asked whether these antibodies impact sporozoite infectivity. To test this, we had to tease apart the relative contribution of antibody in the skin versus antibody in the circulation to the overall inhibition of sporozoite infectivity. Thus, we established a system by which sporozoites delivered by mosquito bite resulted in the same liver parasite burden as those delivered intravenously (i.v.). To do this, we determined the liver parasite burden after a dose range of i.v.-inoculated sporozoites and various numbers of infected mosquito bites and found that the bites of 8 infected mosquitoes resulted in liver parasite burdens that were not significantly different from mice that received i.v. inoculation of 250 to 500 sporozoites (Fig. S5). Using this system, any additional inhibitory effect observed on sporozoites delivered by mosquito bite compared to those inoculated i.v. could be attributed to the activity of antibody at the inoculation site.

10.1128/mBio.02194-18.5 FIG S5 Titration of intravenous sporozoite inoculum to determine an inoculum resulting in a liver-stage infection equivalent to that generated by 8 infected mosquito bites. The indicated doses of *P. berghei* ANKA sporozoites were inoculated i.v., and one group of mice was infected by the bites of 8 infected mosquitoes. Livers were harvested 40 h later and the parasite burden determined by RT-qPCR. There were 4 to 5 mice per group, and horizontal lines represent the mean for each group. Download FIG S5, DOCX file, 0.04 MB. Copyright © 2018 Flores-Garcia et al. 2018 Flores-Garcia et al. This content is distributed under the terms of the Creative Commons Attribution 4.0 International license.

**FIG 6:**
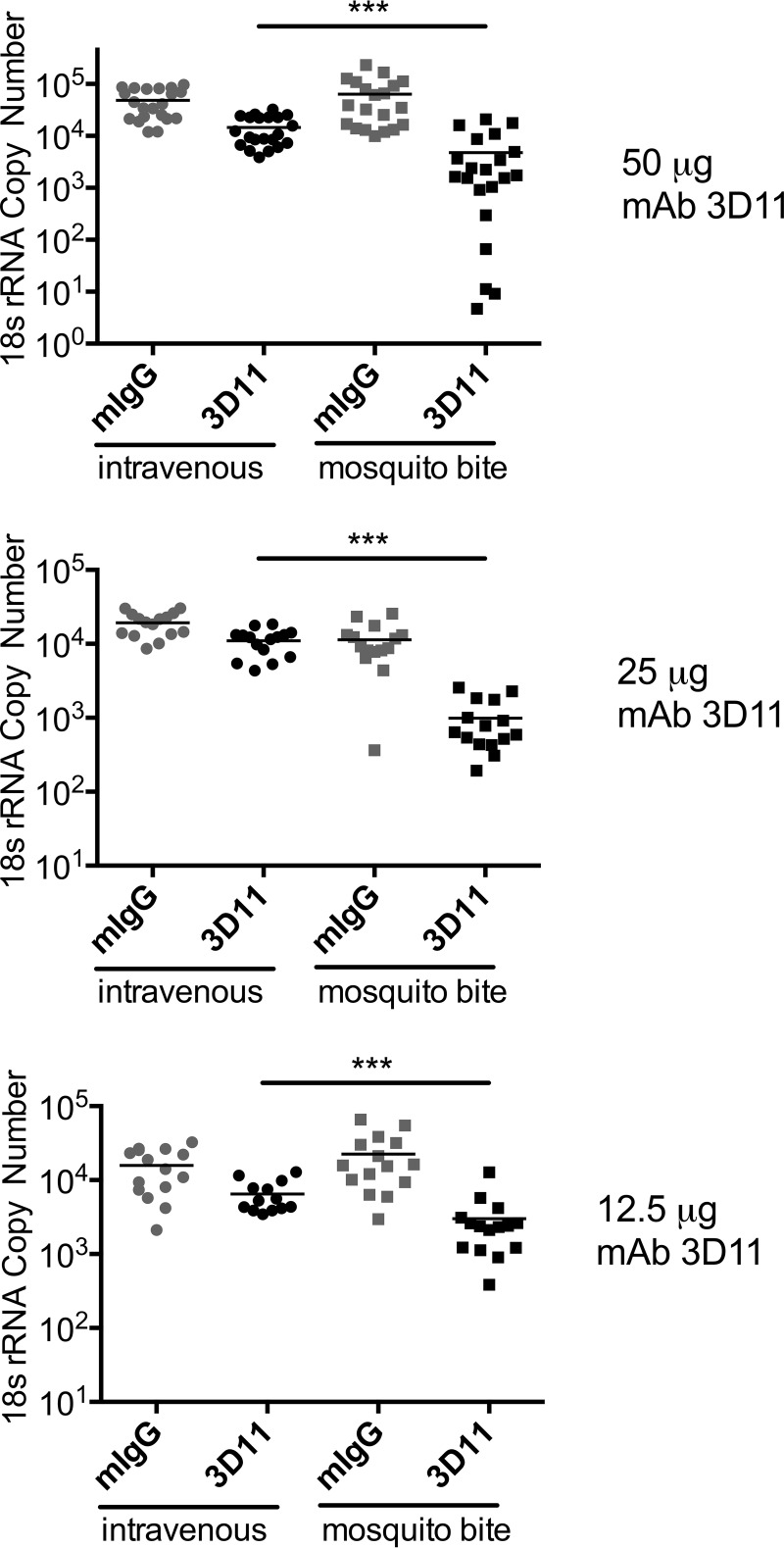
Antibody has greater inhibitory activity in mice infected by mosquito bites. Mice were passively immunized by i.v. inoculation of 50, 25, or 12.5 µg MAb 3D11 and challenged 24 h later with 250 to 500 *P. berghei* sporozoites inoculated i.v. or by 8 infected mosquito bites. Control groups received equivalent doses of mouse IgG and were challenged in the same manner. Forty hours post-sporozoite inoculation, livers were harvested, and the parasite burden was quantified by RT-qPCR. Data were pooled from 3 to 4 independent experiments, with 4 to 6 mice/group per experiment. As shown, the difference between the passively immunized groups challenged with i.v. versus mosquito bites is statistically significant for each concentration (***, *P* < 0.001). The difference between the control groups (mIgG, i.v. versus mosquito bites [MB]) is not statistically significant for the 50-µg or 12.5-µg groups but is significant for the 25-µg group (*, *P* < 0.05). There were no statistically significant differences in the inhibition observed between the 50-, 25-, and 12.5-µg MAb 3D11 groups, challenged either by i.v. or MB inoculation of sporozoites. Statistical comparisons were performed using a linear mixed-effects model on the pooled data.

To test the impact of antibody at the inoculation site, mice were passively immunized with 50, 25, and 12.5 µg of MAb 3D11 or control antibody (mouse IgG [mIgG]), administered intravenously 16 to 24 h prior to sporozoite challenge. Sporozoite challenge was either by i.v. inoculation or mosquito bite, as per the protocol outlined above. Forty hours later, mice were euthanized and livers harvested for quantification of liver parasite burden. The results from these experiments are shown in Fig. 6, where raw data from 3 to 4 experiments per antibody dose are combined. Importantly, in all experiments, controls infected by i.v. inoculation and controls infected by mosquito bite had similar liver parasite burdens; there was no statistically significant difference between the two control groups in the 50- and 12.5-µg groups, and a borderline significant difference between these controls in the 25-µg group (*P* < 0.05). Passive immunization with all three doses of MAb 3D11 had a statistically significant inhibitory activity on i.v.-inoculated sporozoites compared to controls (Fig. 6, mIgG i.v. versus 3D11 i.v.; *P* < 0.01 for 12.5-μg and 50-μg doses, and *P* < 0.05 for 25-μg dose). This is not unexpected, as the majority of previous studies examining the inhibitory activity of CSP-specific antibodies were performed with i.v.-inoculated sporozoites (20, 28, 29), and it would be expected that antibody binds to circulating sporozoites and impacts their ability to enter hepatocytes. To determine if the antibody’s inhibitory activity was enhanced when sporozoites were administered by their natural route of inoculation, we challenged groups of passively immunized mice by infected mosquito bites. As shown in Fig. 6, for all concentrations of passively administered MAb 3D11, protection against mosquito bite-inoculated sporozoites was significantly more pronounced than protection against sporozoites inoculated i.v. (Fig. 6, 3D11 i.v. versus 3D11 mosquito bite; *P* < 0.001 for all MAb 3D11 doses).

**FIG 7:**
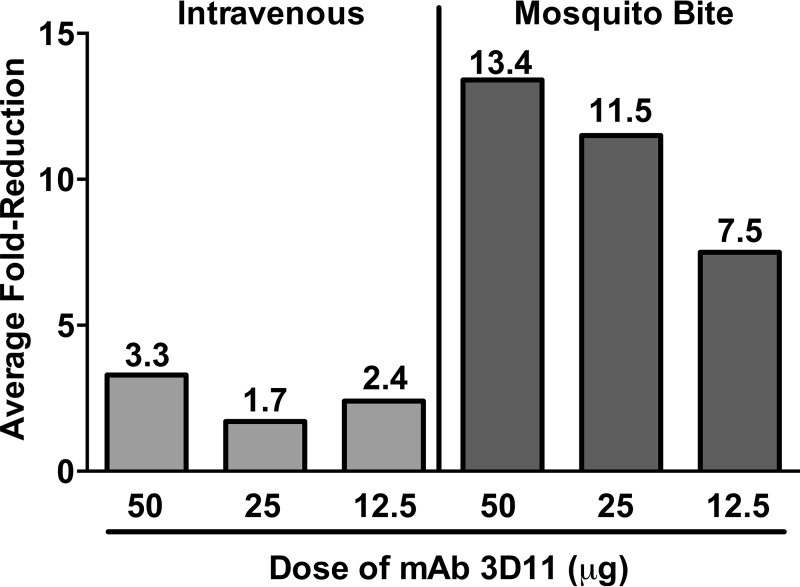
Comparative efficacy of MAb 3D11 after intravenous or mosquito-delivered sporozoite challenge. Data from experiments shown in Fig. 6 were analyzed to calculate the average fold reduction in the 3D11 groups compared to the mIgG controls, for i.v.-inoculated sporozoites (left) and mosquito-inoculated sporozoites (right). Numbers above each bar indicate the average fold reduction for each group. Average fold reduction was calculated by taking the negative inverse of the ratio [−1/(3D11:mIgG)] of the average *P. berghei* 18S rRNA copies in the 3D11 and mIgG groups for each concentration of MAb 3D11. Data from four independent experiments for 50 µg MAb 3D11 (*n* = 20 to 21 mice per group) and three independent experiments each for 25 µg (*n* = 15 mice per group) and 12.5 µg MAb 3D11 (*n* = 13 to 15 mice per group) were used to calculate the average liver parasite burden for each group.

To better compare the efficacy of antibody on sporozoite challenge by i.v. and mosquito bite inoculation, we calculated the average fold change in parasite liver burden in the treatment groups compared to their respective control groups (Fig. 7). When sporozoites are inoculated i.v., MAb 3D11 had a 1.7- to 3.3-fold inhibitory effect on sporozoite infectivity. In contrast, the infectivity of sporozoites inoculated by mosquito bite was inhibited 7.5- to 13.4-fold and in a dose-dependent manner (Fig. 7).

## DISCUSSION

Though it has long been known that antibodies targeting sporozoites can inhibit malaria infection, there is limited knowledge about the mechanism(s) by which they work. In addition, it has generally been thought that these antibodies are working in the bloodstream, an assumption based on the misconception that mosquitoes inoculate sporozoites directly into the blood circulation. In this study, using the rodent malaria model, we show that antibodies specific for CSP work, at least in part, by impacting sporozoite motility at the dermal inoculation site. These findings are significant because they highlight the skin as an important location for antibody-mediated inhibition of sporozoites and motility as a critical sporozoite function that can be the target of protective antibodies.

That antibodies act at the inoculation site is not all together surprising. Studies from the past 10 years have shown that sporozoites are inoculated into the skin and spend minutes to hours there prior to successfully entering the blood circulation (5, 8, 9, 13). Once in the circulation, within minutes, sporozoites go to the liver, where they enter hepatocytes (16, 17). Importantly, there are no data demonstrating that antibody-dependent cell-mediated cytotoxicity occurs in liver-stage parasites, suggesting that by becoming intracellular, sporozoites are protected from antibodies. Thus, the skin is the location where sporozoites are extracellular and vulnerable to antibody-mediated inhibition for a more significant time period. Indeed, a previous study which quantified the infectivity of Plasmodium yoelii administered by mosquito bite or i.v. inoculation in mice passively immunized with a P. yoelii CSP antibody suggested that this might be the case (30). However, interpretation of the data in this study is somewhat confounded by the significantly lower liver loads in mice infected by mosquito bite than those in mice infected by i.v. inoculated sporozoites, making it difficult to ascertain whether the greater impact of antibody was due to a lower sporozoite load or to its enhanced activity on mosquito-inoculated sporozoites. Here, we established an infection assay that gave approximately equivalent liver loads after i.v. inoculation of sporozoites and mosquito inoculation of sporozoites and found that in the presence of antibody, infectivity is significantly more attenuated in mice infected by mosquito bite than with i.v. inoculation. Indeed, 50 and 25 μg of antibody specific for the CSP repeats decrease sporozoite infectivity by over 10-fold when they are inoculated by mosquito versus 2- to 3-fold when they are inoculated intravenously. These data suggest that the impact of antibody at the inoculation site may be especially important at lower antibody concentrations that do not have a significant impact on sporozoites in the blood circulation. Moving forward, our data suggest that antibodies targeting migratory sporozoites at the inoculation site could be more effective than targeting the infectious sporozoites found in the liver, where the time frame for inhibition is significantly shorter. Furthermore, these data highlight the need to perform parasite challenge in the skin for the evaluation of protective immunity.

We also demonstrate that an important mechanism by which CSP repeat antibodies act at the inoculation site is via their impact on sporozoite motility. We observed that antibody specific for the CSP repeats decreases the percentage of parasites that are motile, with the remaining motile parasites exhibiting reduced displacement and speed. Interestingly, the greatest impact of antibody was on those sporozoites moving farthest and fastest, suggesting that it may be these sporozoites that disproportionately succeed in entering blood vessels. Supporting this hypothesis, we did find significant inhibition of blood vessel invasion in passively immunized mice. These results suggest that sporozoites with high displacements may be more likely to find and enter blood vessels, consistent with previous data demonstrating that sporozoite dispersal early after their inoculation maximizes the tissue volume they explore, which may be important in locating blood vessels (7). Though the precise relationship between decreases in displacement and inhibition of blood vessel entry to degree of inhibition of sporozoite infectivity remains unclear, it would be an important area of investigation for future study. Previous *in vitro* studies and one *in vivo* study using supraphysiologic concentrations of antibody found that antibodies specific for the CSP repeats can immobilize sporozoites (23, 31). Here, we show that at titers more in line with those found in immunized individuals, antibodies specific for the CSP repeats have more subtle effects on motility, and these effects can be associated with a significant impact on infection. Importantly, these *in vivo* data could be used to establish parameters in the rodent model for screening vaccine candidates targeting the migratory sporozoite.

Importantly, we cannot conclude that the impact of antibody on sporozoite motility in the skin is the only mechanism by which antibody acts to inhibit sporozoite infection. As shown both in this study and many previous studies, when sporozoites are injected intravenously in mice with circulating anti-sporozoite antibodies, there is also a significant inhibition of parasite infection in the liver, and this suggests an effect of antibodies on the migration of sporozoites to the liver parenchyma, which also requires parasite motility. Importantly, early studies with MAb 3D11 indicated that Fab monomers *in vivo* had a protective effect comparable to that observed with intact MAb 3D11 IgG1 (28). This indicates that Fc functions are not needed for the protective activity of this antibody. Nevertheless, we cannot rule out that protection conferred by other antibodies requires complement fixation, leading to sporozoite destruction via direct lysis or phagocytosis. Additionally, it remains to be determined whether Fc-mediated mechanisms found *in vitro* are relevant to protection observed *in vivo*. Our current experiments suggest that *in vivo*, a direct effect of antibody on sporozoite motility at the inoculation site is one of the mechanisms by which antibody protects.

Our findings may be relevant to the malaria vaccine effort targeting preerythrocytic stages of Plasmodium species. To date, RTS,S, a subunit vaccine based on CSP, is the only malaria vaccine candidate to show efficacy in phase III clinical trials, conferring 50% protection in preventing malaria and 45% efficacy in preventing severe disease (32). Nonetheless, the efficacy of RTS,S wanes significantly after 1 year (33) and falls short of community-established benchmarks, indicating that more work is needed to produce a fully efficacious vaccine. Follow-up studies on RTS,S vaccinees suggest that antibody titers specific for the CSP repeat region correlate with protection (2–4). Our data raise the possibility that the partial efficacy of RTS,S is due to the impact of antibody on sporozoite motility at the inoculation site. Further studies with sera from protected and unprotected RTS.S-immunized volunteers are needed to determine how protective antibodies are functioning. *In vivo* studies in mice with P. berghei sporozoites expressing P. falciparum CSP could be employed to elucidate whether, as we observed in our studies, antibodies from RTS,S-immunized volunteers are functioning by impacting sporozoite motility at the inoculation site.

## MATERIALS AND METHODS

### Animals.

Five- to 8-week-old female C57BL/6nTac and C57BL/6j mice were purchased from Taconic Farms (Derwood, MD) and Charles River Laboratories (Frederick, MD), respectively, and housed in the animal facility at the Johns Hopkins Bloomberg School of Public Health. C57BL/6j mice were used for all imaging studies, and C57BL/6nTac mice were used for all infectivity studies. For all experiments, mice in the control and experimental groups were age and sex matched. All animal work was performed in accordance with the Institutional Animal Care and Use Committee guidelines (protocols M017H325 and M016H35).

### Parasites and mosquitoes.

Sporozoite infectivity studies were performed with wild-type P. berghei ANKA strain parasites. Imaging studies were performed with P. berghei ANKA strain parasites expressing mCherry under the *uis4* promoter (PBANKA_0501200) previously described by Hopp et al. (7). For some of the imaging studies, P. berghei parasites expressing P. falciparum CSP were used. These were generated in the mCherry line outlined above, and the methodology for their generation and verification can be found below and in Fig. S1. Anopheles stephensi mosquitoes were reared in the insectary at the Johns Hopkins Bloomberg School of Public Health, infected with the indicated parasites, and used for experiments between days 18 and 22 postinfectious blood meal.

### Generation of a P. berghei mCherry parasite expressing P. falciparum CSP.

P. berghei parasites expressing mCherry (7) were used to generate transgenic parasites expressing P. falciparum CSP. Transfections were performed as described by Janse et al. (34), using pR-CSPfFL containing the P. falciparum *csp* coding region with a P. berghei signal sequence, the hDHFR selection cassette, and *csp* 5′ and 3′ untranslated regions (UTRs) (35). pR-CSPfFL was cut with XhoI and KasI and transfected into schizont cultures of mCherry P. berghei parasites by electroporation using an Amaxa Nucleofector. Following transfection, parasites were inoculated into Swiss Webster mice and selected with pyrimethamine. Parental populations of parasites were cloned by limiting dilution in mice, and clones were verified by PCR and sequencing.

### Confocal microscopy and cell tracking.

Sporozoites were imaged in the ear pinna of mice, as previously described (6, 7). Mice were anesthetized by intraperitoneal injection of ketamine (35 to 50 mg/kg body weight) and xylazine-hydrochloride (6 to 10 mg/kg body weight), and P. berghei mCherry sporozoites were injected into the ear at multiple sites in volumes of 0.5 to 2 μl, using a NanoFil 10-μl syringe with an NF33BV-2 needle (World Precision Instruments). Mice were kept in a microscope chamber warmed to 28°C, and their ears were fixed onto a cover glass and imaged with a 10× objective on an inverted Zeiss Axio Observer Z1 microscope with a Yokogawa CSU22 spinning disk. Ten minutes following intradermal inoculation of sporozoites, 5-min videos were recorded, with each Z-stack consisting of 3 slices spanning a total depth of 30 to 50 µm, with a frame rate of 1,000 ms/frame, using an electron-multiplying charge-coupled-device (EMCCD) camera (Photometrics, Tucson, AZ, USA) and the 3i SlideBook 5.0 software. Videos were analyzed using Fiji and the ICY 1.8.6.0 softwares, which are freely available from https://fiji.sc and http://icy.bioimageanalysis.org (36), respectively. Speed and net displacement were determined by automated tracking of sporozoites from recorded videos, while the percentage of total sporozoites that were motile and nonmotile and the number that exhibited circular or meandering motility were manually counted. Blood vessel invasion was also quantified manually and scored when a rapid change in sporozoite speed was observed as previously outlined (7). For all intravital imaging experiments, each biological replicate was performed with two mice, one immunized and one naive.

### Immunization of mice.

For immunization with irradiated sporozoites, 6- to 7-week-old female C57BL/6j mice were immunized intravenously with 10,000 γ-attenuated sporozoites 3 times at 2-week intervals with either wild-type P. berghei parasites or transgenic P. berghei parasites expressing full-length P. falciparum CSP. Mice immunized with this regimen were protected from sporozoite challenge. Three weeks following the final immunization, mice were used for confocal microscopy to investigate sporozoite motility. For passive immunization experiments, the indicated concentration of MAb 3D11 (20), an IgG1 subclass, was inoculated intravenously 16 to 24 h prior to the inoculation of sporozoites.

### Infectivity experiments.

For each experiment, the same cage of P. berghei ANKA-infected Anopheles stephensi mosquitoes was used to infect mice by i.v. or mosquito bite inoculation. The prevalence and intensity of infection in the cage were determined on day 18 to 22 postinfectious blood meal by microscopically examining the salivary gland sporozoite load from each of 20 mosquitoes. Mosquitoes were only used if the average sporozoite load per mosquito was greater than 10,000 sporozoites and the cage had a prevalence of infection of 90% or greater. For intravenous challenge, infected mosquitoes were dissected in cold Leibovitz’s L-15 medium (catalog no. 11415064; Thermo Fisher Scientific), and sporozoites were counted using a hemocytometer. A working solution with 250 to 500 sporozoites per 200 μl was prepared in RPMI 1640 and inoculated into mice through the tail vein. For mosquito bite challenge, infected mosquitoes were anesthetized on ice and transferred to feeding tubes 1 day prior to challenge. No more than four mosquitoes were placed in a single tube. Mosquitoes were sugar-starved overnight but provided with water until ∼6 h prior to mosquito bite challenge. Female C57BL/6nTac mice were anesthetized with intraperitoneal injection of ketamine (35 to 50 mg/kg body weight) and xylazine-hydrochloride (6 to 10 mg/kg body weight) and placed on a warming plate set to 37°C. Each mouse was exposed to 8 mosquito bites, 4 bites per ear. Visualization of the biting process ensured that each mouse was probed upon by 8 mosquitoes, and if a mosquito did not probe, it was replaced by another mosquito.

### Quantification of parasite liver burden by RT-qPCR.

Parasite liver burden was measured by quantitative reverse transcription-PCR (RT-qPCR), as outlined by Bruña-Romero et al. (37). Briefly, 40 h after parasite challenge, mice were sacrificed, and their livers were harvested, washed twice in cold phosphate-buffered saline (pH 7.4), weighed, and homogenized in 10 ml of prechilled Tri-Reagent (Molecular Research Center, Inc., Cincinnati, OH) for 1 min at full speed using a homogenizer (Kinematica, Bohemia, NY). RNA was extracted from 1 ml of the liver homogenate by phenol-chloroform extraction, and the concentration was measured using a NanoDrop spectrophotometer (Thermo Fisher Scientific). RNA (1.5 µg) was reverse transcribed using random hexamers (Thermo Fisher Scientific) and the following cycling profile: 25°C (10 min), 42°C (20 min), 95°C (5 min), and 5°C (5 min). Following this, quantitative PCR (qPCR) was performed using primers specific for the P. berghei 18S rRNA gene, as previously described by Kumar et al. (38). Quantification of P. berghei 18S rRNA copy number was based on a standard curve prepared using 10-fold serial dilutions (10^7^ to 10^2^) of the P. berghei 18S rRNA gene.

### Statistical analysis.

Statistical comparisons of motility-based parameters for Fig. 1, 4, S2, and S4 were performed using a linear mixed-effects model on the pooled data, accounting for experimental variation among biological replicates as a random effect using STATA version 13.0 software. Since the distribution of sporozoite net displacement in both naive and immunized mice was right-skewed, the data were square root-transformed prior to analysis. Comparisons of motility type between immunized and naive groups in Fig. 3B were performed using nonparametric Mann-Whitney tests (two-tailed, α = 0.05) with the GraphPad Prism 6 software. Statistical analysis of the proportion of motile and nonmotile sporozoites in naive and immunized mice (Fig. 1A, 3A, and 4A) was performed using Fisher’s exact test. Statistical comparisons of P. berghei 18S rRNA copy number between control and passively immunized mice in Fig. 6 were performed using a linear multilevel mixed-effects model, accounting for experimental variation and interaction of treatment and route, using the STATA version 13.0 software.
