# Supplementary material for: Antibody-Mediated Protection against Plasmodium Sporozoites Begins at the Dermal Inoculation Site
Source: mBio. 2018 Nov 20;9(6):e02194-18. doi: 10.1128/mBio.02194-18 (PMC6247089; doi:10.1128/mBio.02194-18)
Supplement: FIG S3 [file mbo006184170sf3.docx]

Supplemental Figure 3

**E**

**D**

**B**

**A**

| **Parasite** | **Salivary Gland Sporozoites** |
| --- | --- |
| Pb | 14,233 |
| PbPfCSP | 15,133 |

**C**
