# Supplementary material for: Antibody-Mediated Protection against Plasmodium Sporozoites Begins at the Dermal Inoculation Site
Source: mBio. 2018 Nov 20;9(6):e02194-18. doi: 10.1128/mBio.02194-18 (PMC6247089; doi:10.1128/mBio.02194-18)
Supplement: FIG S5 [file mbo006184170sf5.docx]

Supplemental Figure 5
